# Collagen from Bovine Omentum: Extraction and Characterization

**DOI:** 10.3390/foods15010044

**Published:** 2025-12-23

**Authors:** Ajay Mittal, Catherine Collins, Lena Madden, Nigel Brunton

**Affiliations:** 1 UCD Institute of Food and Health, University College Dublin, Belfield Campus, D04 V1W8 Dublin, Ireland; ajay.mittal@ucd.ie; 2 LIFE Institute, Technological University of the Shannon, V94 EC5T Limerick, Ireland; catherine.collins@tus.ie (C.C.); lena.madden@tus.ie (L.M.)

**Keywords:** bovine omentum, acid-soluble collagen, Protana^®^ Prime, techno-functional properties, collagen characterization

## Abstract

Bovine omentum, a by-product of beef processing, offers potential for collagen recovery within the circular bioeconomy. It consists mainly of lipids (42.14%) and proteins (18.79%), such as collagen. In this study, collagen was isolated using acid-based and enzymatic methods. Acid-soluble collagen (ASC) was successfully extracted, yielding 3.98%. Additionally, enzymatic extraction of collagen from the residue obtained after ASC extraction using Protana^®^ Prime (1–10%, *w*/*w*) resulted in variable yields (4.98% to 11.15%) (*p* < 0.05). The maximum solubility of all collagen samples was observed at pH 3, while NaCl concentrations above 4% (*w*/*v*) significantly reduced solubility (*p* < 0.05). ASC demonstrated the highest emulsifying activity index and emulsion stability index (213.73 m^2^/g and 172.09 min, respectively) (*p* < 0.05), whereas enzyme-extracted collagens exhibited comparatively lower emulsifying capacities, particularly at higher enzyme concentrations (7.5% and 10%). FTIR spectra revealed characteristic bands for collagen, indicating that the triple helical structure was maintained, irrespective of treatment. All collagen samples contained glycine as the major amino acid (approximately 1/3rd of the total amino acid) with proline and hydroxyproline. SDS-PAGE identified type I collagen, which consisted of αI and αII chains. Therefore, bovine omentum would be an alternative source of collagen for various applications in the food industry.

## 1. Introduction

In the past years, global meat production and consumption have increased, reaching 362.9 million tons and 44.1 kg per capita consumption, respectively, in 2023 [1]. Specifically, global beef and buffalo production totalled 76.6 million tons, with consumption averaging 9.3 kg per capita [1,2]. This upward trend is expected to persist, driven by population growth and rising income levels worldwide. In 2024, the European Union produced 6.6 million tons of beef, while the United Kingdom produced 934,000 tons, representing year-on-year increases of 3% and 4%, respectively, compared to 2023 [3]. Within Europe, Ireland’s beef sector is a major component of the country’s agricultural economy, characterized by its grass-based production system and high dependence on exports. The total production of Irish beef was estimated as 603,000 tons in 2024 [4]. In 2024, primary Irish beef exports (excluding offal and value-added beef) were valued at €2.8 billion (primary beef: 507,000 tons), an increase of 6%. Beef exports accounted for over 66% of Ireland’s total meat exports, making beef the country’s second most valuable agri-food export category after dairy [5]. Hence, the Irish beef sector plays a vital role in the Irish economy. However, the growth of the meat processing industry has also led to a significant increase in the by-products generation.

**Figure 1:**
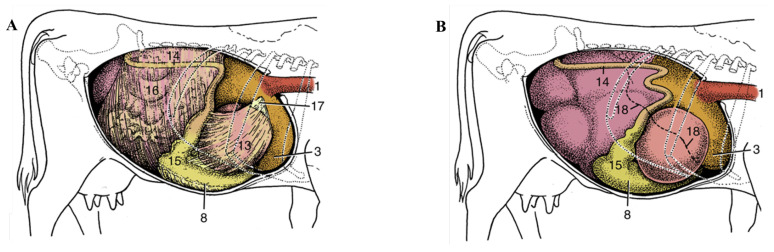
Topography of the bovine abdominal viscera. (**A**) Relationship of abdominal viscera to the right abdominal wall; the liver has been removed. (**B**) Position of the parts of the stomach seen from the right. 1, Oesophagus; 3, reticulum; 8, body of abomasum; 13, omasum, covered by lesser omentum; 14, descending duodenum; 15, pyloric part of abomasum; 16, greater omentum covering the intestinal mass; 17, lesser omentum cut away from the liver; 18, position of caudoventral border of liver. Source: https://pressbooks.umn.edu/largeanimalanatomy/chapter/abdomen-2/ (accessed on 20 October 2025).

The meat slaughtering and processing industry produces solid animal by-products as waste, which present major challenges because of their high organic content and potential to harbour pathogens, and their disposal in landfills can lead to surface water contamination, unpleasant odours, and increased greenhouse gas emissions. In general, solid meat by-products are classified into two main types: edible offal and inedible offal. The term *offal* refers to the portions, such as organs and internal parts, that separate from the carcass during processing. Inedible offal consists of blood, tissue, fat, hides, hooves, horns, bones, and lungs [6]. Inedible offal can be fractionated into value-added products such as blood meal and plasma protein from blood, collagen and gelatine from hides, minerals and bone powder/meal from bones, horns and hooves, often used as soil conditioners and organic fertilizers. This presents an opportunity for the beef industry to develop value-added products and alternate markets for by-products to increase the profitability of beef-processing operations and reduce waste. Moreover, the conversion of by-products to usable biomaterials has great importance in securing the future viability of the beef industry by adopting sustainable practices. Apart from the aforementioned inedible components, the bovine omentum, also known as the butcher’s apron, is of particular interest for further utilisation. The omentum, also known as the epiploon, is a flat layer of adipose tissue covered by visceral peritoneum (Figure 1). It extends downward from the greater curvature of the stomach and drapes over the intraperitoneal organs, primarily covering the small and large intestines [7,8]. In the live ruminant, the omentum supports and holds the abdominal organs in place and has biological functions including lipid storage and immune regulation [9]. After slaughter, the bovine omentum is generally considered an inedible processing by-product, unless it is specifically harvested for culinary or rendering purposes under controlled hygienic conditions. The bovine omentum is mainly utilized as a source of animal fat, which can be rendered into tallow for industrial uses such as soap, cosmetics, and feed production. In addition to its high fat content, the omentum also contains connective tissue rich in collagen fibres. However, to date, we cannot find any reports on the proximate composition of bovine omentum.

Owing to its excellent biocompatibility, biodegradability, and low antigenicity, collagen has become a highly valuable biomaterial for numerous biomedical applications, including tissue engineering, regenerative medicine, and implantable medical devices [10,11]. Moreover, collagen possesses various technological properties such as film forming, foaming, emulsifying, and gelling, and is thus suitable for use in various food products and as an edible packaging material [12,13]. In addition, collagen peptides produced through enzymatic hydrolysis have various biological activities, including antioxidant, ACE-I inhibitory, DPP-IV inhibitory, and anti-freezing activities [14]. Owing to their valuable techno-functional and bioactive properties, the demand for collagen and its derivatives has increased significantly, with the global market projected to reach approximately $6.63 billion by 2025 [15]. Structurally, collagen fibrils are composed of bundles of tropocollagen molecules, each about 300 nm in length and 1.5 nm in diameter [16,17]. The protein is a right-handed triple helix formed by three polypeptide α-chains. Each chain features a repeating tripeptide sequence of (Gly–X–Y)_n_, where X and Y are typically proline or hydroxyproline [18]. Proline helps maintain the helical conformation of the α-chain, while hydroxyproline contributes to the stability of the collagen triple helix through hydrogen bonding via its hydroxyl groups.

Therefore, omentum represents a promising and underutilized source of collagen. To the best of our knowledge, no previous studies have reported the extraction and characterization of collagen from bovine omentum. This gap presents a valuable opportunity for the beef industry to develop value-added products and explore alternative markets for slaughterhouse by-products, thereby enhancing the overall profitability and sustainability of beef-processing operations. Accordingly, the present study aimed to determine the proximate composition, including fatty acid and amino acid profiles of bovine omentum, and to extract, characterize, and determine techno-functional properties of collagen obtained through acid solubilization and enzyme (Protana^®^ Prime) assisted extraction methods. Lipids were characterized based on total lipid content and fatty acid composition, while proteins were characterized by total protein content, amino acid composition, and collagen-specific markers such as hydroxyproline. Structural features of the extracted collagen were further assessed using FTIR spectroscopy and SDS-PAGE.

## 2. Materials and Methods

### 2.1. Chemical Reagents

All chemicals of analytical grade were procured from Thermo Fisher Scientific, Kandel, Germany. Protana^®^ Prime, an exopeptidase (1067 LAPU/g), was supplied by Novonesis, Bagsværd, Denmark. Amino acid standard mix (AAS18), along with tryptophan, hydroxyproline, asparagine, and glutamine of HPLC grade, along with o-phthaldialdehyde (OPA) and 3-mercaptoethanol, were procured from Sigma Aldrich Ireland Limited, Arklow, Co. Wicklow, Ireland. HPLC-grade methanol and acetonitrile were obtained from Fisher Scientific Ireland, Dublin, Ireland.

### 2.2. Bovine Omentum Preparation

Bovine omentum was supplied by the Irish red meat processor. The sample was sealed in a polyethylene bag after slaughtering and kept at −20 °C. The sample was transported in frozen form to the lab; thereafter, it was cut into 5 × 5 cm pieces, vacuum-sealed, and returned to −20 °C storage until further use.

### 2.3. Determination of Proximate Composition, Amino Acid Composition, and Fatty Acid Profile of Bovine Omentum

#### 2.3.1. Proximate Analysis

Moisture, crude protein, crude lipid, and ash content were determined according to AOAC’s official Method of Analysis [19]. Carbohydrate content was calculated by difference as follows:

%Carbohydrate=100−(%Moisture+%Crudelipid+%Crudeprotein+%Ash)

#### 2.3.2. Fatty Acid Analysis

Fatty acid methyl esters (FAMEs) using 1.5 g of bovine omentum were prepared using the rapid microwave-assisted method as described by Brunton, et al. [20]. FAMEs separation using CP-Sil 88 capillary column (100 m × 0.25 mm i.d., 0.2 μm) was performed on a GC equipped with a flame ionisation detector (Agilent Technologies, Santa Clara, CA, USA) at parameters suggested by Brunton, Mason and Collins Jr [20]. Fatty acids were identified by comparing their retention times with the Supelco 37-component FAME mix. The data acquisition was performed using OpenLAB CDS 2.1 (Agilent Technologies, CA, USA). FAMEs were quantified based on the IS method. The fatty acid content was expressed in mg/g wet weight.

#### 2.3.3. Amino Acid (AA) Analysis

AA analysis was carried out using an in-house method which utilised microwave-assisted digestion of the protein coupled to HPLC-UV with pre-column derivatization [21,22]. For microwave-assisted hydrolysis, 1 g of bovine omentum was weighed into a MARSXpress PFA reaction vessel, and then 8 mL of 6 M HCl containing phenol (1%, *w*/*v*) was added to the vessel. Each open vessel was then flushed with nitrogen for 30 s to displace air and immediately sealed with a cap before being placed in the microwave digestion system (CEM MARS 6^TM^, CEM Corporation, Matthews, NC, USA). The digestion was conducted at 1000 W at 155 °C for 1 h with stirring to ensure complete protein hydrolysis, following the manufacturer’s (CEM) recommended conditions for microwave-assisted acid digestion. The vessels were allowed to cool at room temperature after completion of digestion. Following cooling, the pH of the hydrolysate was adjusted to between 6.5 and 7 using NaOH (1 N or 10 N), transferred into a 25 mL volumetric flask, and the volume was made up using deionized water. Pre-column derivatization was carried out at room temperature by mixing 20 µL of the hydrolysate with 10 µL of 0.4 N borate buffer and 10 µL of FMOC-Cl reagent (2.5 mg/mL in acetonitrile) in an Eppendorf tube by vortexing for 1 min. Subsequently, 30 µL of OPA reagent (50 mg of OPA in 1.25 mL of methanol with 50 μL of 3-mercaptoethanol and 11.2 mL of 40 mM borate buffer at pH 10.2) was added, and then the mixture was vortexed for an additional 2 min. Finally, derivatized amino acids were transferred to a vial and subjected to chromatographic separation. The separation of derivatives was performed using a Zorbax Eclipse AAA column (150 × 4.6 mm i.d., 5 µm) at a constant temperature of 40 °C on an HPLC (Agilent Technologies, Palo Alto, CA, USA) equipped with a UV detector. Sodium phosphate buffer (40 mM; pH 7.8) as mobile phase A and methanol–acetonitrile–water (45:45:10, *v*/*v*/*v*; mobile phase B) were used at a flow rate of 2.0 mL/min. The linear separation gradient was as follows: 0 min: 0% B; 1.9 min: 0% B; 18.1 min: 57% B; 18.6 min: 100% B; 24.3 min: 100% B; 25.2 min: 0% B; and 26.0 min: 0% B. The total run time was 26 min, which included the column regeneration and returning to initial conditions. The detection of amino acids took place at 338 nm and 262 nm. Agilent OpenLab CDS 2.5 software was used for instrumental control and data acquisition. Identification and quantification of amino acids were carried out by comparing their retention time and calibration curves obtained from the amino acid mix standard under similar conditions. The amino acid content was expressed as mg/g wet weight.

### 2.4. Extraction of Collagen from Bovine Omentum

Bovine omentum (40 g) was thawed under running water and manually cut into 1 × 1 cm pieces. The pieces were then treated with a solution containing NaOH (0.5 M) and NaCl (0.5 M) at a sample-to-solution ratio of 1:20 (*w*/*v*) for 24 h with continuous stirring using an overhead stirrer at room temperature (20–22 °C) to remove non-collagenous protein. After the alkaline treatment, the samples were thoroughly washed with water until the pH was neutral or faintly basic. The treated samples were then defatted using 30% isopropanol at a solid-to-solvent ratio of 1:20 (*w*/*v*) for 24 h. Following defatting, the samples were washed three times with 10 volumes of water. Thereafter, defatted samples were stirred in acetic acid (0.5 M) at a solid-to-solvent ratio of 1:50 (*w*/*v*) for 48 h. The resulting mixture was filtered through two layers of cheesecloth, and the filtrate was collected and subjected to isoelectric precipitation at a pH between 5 and 5.5 using 2 N and 6 N NaOH for acid-soluble collagen (ASC).

The residue obtained after ASC extraction was subjected to homogenization in acetic acid (1 M). The sample-to-solution ratio of 1:50 (*w*/*v*) was maintained, and the final pH of the mixture was adjusted to 3.5 using 2 N NaOH. Thereafter, Protana^®^ Prime at varying concentrations (1–10%, *w*/*w* of wet sample) was added, and the mixture was stirred using an overhead stirrer for 24 h at room temperature. The mixture was filtered through two layers of cheesecloth, and the filtrate was collected for isoelectric precipitation to obtain enzyme-aided extracted collagen. Following precipitation, the residual supernatant was collected for further analysis.

### 2.5. Analyses of Extracted Collagen

#### 2.5.1. Yield

The yield was calculated using the following equation:

$$Yield\left(\%\right)=\frac{Dry\;weight\;of\;extracted\;collagen\;(g)}{Wet\;weight\;of\;bovine\;omentum\;(g)}\times 100$$

#### 2.5.2. Degree of Hydrolysis (DH), Free Amino Acid Content, and Protein Content

The residual supernatant remaining after the precipitation of collagen, obtained through enzymatic extraction, was used for analysis. DH was determined using OPA according to the method described by Nielsen, et al. [23]. The α-amino content quantified was determined using TNBS according to the method of Benjakul and Morrissey [24]. The protein content of the supernatant was determined using the Biuret method [25].

#### 2.5.3. Hydroxyproline Content

Hydroxyproline content was determined using HPLC equipped with a UV detector with some modifications [26]. The sample of approximately 0.5 g was subjected to microwave-assisted hydrolysis as described in Section 2.3.3. Then, the pH of the hydrolysate was adjusted to between 6.5 and 7 using NaOH (1 N and 10 N), transferred into a 25 mL volumetric flask, and the volume was made up with deionized water. For derivatization, 60 µL of 0.4 N borate buffer was added to an Eppendorf tube, followed by the addition of 20 µL of filtered hydrolysate. The mixture was vortexed immediately for 5 s. Then, the mixture was derivatized by adding 40 µL of FMOC-Cl (2.5 mg/mL in acetonitrile) and vortexing again for 1 min. The mixture was transferred into an HPLC vial before chromatographic separation. HPLC separation with UV detection was carried out using the conditions described in Section 2.3.3 at a wavelength of 262 nm. L-hydroxyproline as a standard was used to determine the retention time and to make a standard curve (112.5–1800 pmol/µL). The standard curve equation was obtained through linear regression data analysis. The hydroxyproline content was reported as mg/g sample.

#### 2.5.4. Determination of Salt and pH Solubility of Collagen Samples

Salt solubility of collagen samples was determined as suggested by Zeng, et al. [27] with slight changes. In brief, 10 mL of sample (6 mg/mL) in acetic acid (0.5 M) was mixed with 10 mL of NaCl at various concentrations (0% to 18%, *w*/*v*) for 30 min at room temperature, followed by centrifuging at 20,000× *g* for 30 min. The pH solubility method was adopted from Shaik, et al. [28] with some modifications. Briefly, 15 mL of sample (10 mg/mL) was added to a beaker, and then the pH was adjusted using either 2 M or 6 M HCl and 2 M or 6 M NaOH from 3 to 12. The solution was stirred for 30 min at room temperature. Thereafter, the volume of the solution was made up to 25 mL by adding distilled water. The solutions were again stirred for 30 min and then centrifuged as described above. Finally, protein content in the supernatant was determined using the Lowry method [29]. Relative salt and pH solubility was calculated according to the following formula:

$$Relative\;solubility\left(\%\right)=\frac{Protein\;content\;in\;supernatant\;(mg/mL)}{Initial\;protein\;content\;(mg/mL)}\times 100$$

#### 2.5.5. Emulsifying Properties

Samples (10 mg/mL) were dissolved in acetic acid (0.5 M). A sample (40 mL) with sunflower oil (10 mL) was homogenized at 20,000 rpm for 1 min. Then, aliquots (100 µL) from the bottom of the tubes were taken at 0 and 30 min and diluted 50 times using acetic acid (0.5 M). The absorbance was read using a spectrophotometer at 500 nm. Emulsion activity index (EAI) and emulsifying activity index (ESI) were calculated as follows:

$$EAI\left(m^{2}/g\right)=\frac{2\times 2.303\times A_{0}\times DF}{C\times L\times \left(1-\emptyset \right)\times 10,000}$$

$$ESI\left(min\right)=A_{0}\times ∆t/{(A}_{0}-A_{30})$$

DF: 50; Φ: 0.20; C: concentration of protein (g/mL); L: path length (1 cm); A_0_: OD_500_ at 0 min; A_30_: OD_500_ at 30 min; Δt: 30.

#### 2.5.6. FTIR Analysis of Collagen Samples

Before analysis, the samples were kept in a desiccator containing P_2_O_5_ for 3 days to absorb free moisture. FTIR spectra of dehydrated samples were obtained Bruker Invenio S FTIR spectrometer equipped with A225/Q Platinum unit with a single reflection diamond crystal (Bruker OPTIK GmbH, Ettingen, Germany). The sample (approximately 25 mg) was put on an ATR cell using a spatula. Afterwards, the sample was scanned at a speed of 0.2 cm/s, 32 scans, 4 cm^−1^ of the resolution, and a spectral range from 4000 to 400 cm^−1^. The data were analyzed using Opus 8.5 software.

#### 2.5.7. Amino Acid Composition of Extracted Collagen Samples

Amino acid composition of collagen samples was determined as described in Section 2.3.3. The amino acid content and composition were reported as mg/g dry weight and amino acid residues/1000 amino acid residues, respectively.

#### 2.5.8. Protein Patterns of Collagen Samples

Sodium dodecyl sulphate-polyacrylamide gel electrophoresis (SDS-PAGE) of collagen samples was performed for protein patterns [30]. Samples (20 μg of protein) were loaded onto a polyacrylamide gel (4% stacking and 7.5% running gel) and separated using a constant current of 15 mA/gel. Gel staining and destaining were performed using Coomassie blue R-250 (0.05%, *w*/*v* in 15% (*v*/*v*) methanol and 5% (*v*/*v*) acetic acid) and a mixture of methanol (30%, *v*/*v*) and acetic acid (10%, *v*/*v*). Intensity of protein bands was quantified using public domain digital analysis software (ImageJ 1.42q, Bethesda, MD, USA).

### 2.6. Statistical Analysis

The study employed a completely randomized design (CRD). All experiments and statistical analyses were carried out in triplicate (*n* = 3). Data were subjected to analysis of variance (ANOVA), and mean comparisons among treatments were conducted using Duncan’s multiple range test at a significance level of *p* < 0.05. Statistical analyses were performed using SPSS software (SPSS for Windows, Version 28; SPSS Inc., Chicago, IL, USA).

## 3. Results and Discussion

### 3.1. Proximate Composition, Fatty Acid Profile, and Amino Acid Profile of Bovine Omentum

The proximate composition of bovine omentum on a wet basis is given in Table 1. It contains a high crude lipid content of 42.14%, followed by 35.25% moisture and 18.79% crude protein. Bovine omentum is composed of adipose tissue, which serves as a storage site for fat [7,9]. In contrast, carbohydrates (3.77%) and ash (0.06%) were found at relatively low levels.

The amino acid and fatty acid compositions of the bovine omentum sample are presented in Table 2. The total amino acid (TAA) content was 202.98 mg/g wet weight, comprising 70.04 mg/g wet weight essential amino acids (EAA) and 131.42 mg/g wet weight non-essential amino acids (NEAA). Among the amino acids, glutamic acid (22.41 mg/g wet weight), phenylalanine (22.80 mg/g wet weight), alanine (22.29 mg/g wet weight), and glycine (43.10 mg/g wet weight) were the most abundant components. Moreover, hydroxyproline (10.83 mg/g wet weight) and proline (12.81 mg/g wet weight) were also present in significant amounts. Although no previous studies have reported the amino acid composition of bovine omentum, the detection of hydroxyproline strongly suggests a substantial presence of structural proteins such as collagen and elastin, as hydroxyproline is found exclusively in these proteins [31]. Similarly, collagen extracted from ox-hide and calf skin has been shown to contain hydroxyproline [32]. Generally, glycine, proline, and hydroxyproline contribute more than half of the total amino acids in collagen. Therefore, the presence of glycine, alanine, hydroxyproline, and proline in the bovine omentum suggests a substantial presence of structural proteins, particularly collagen-related proteins.

Fatty acid analysis revealed that oleic acid was the predominant fatty acid, followed by palmitic and stearic acids. Unsaturated fatty acids were 44% of total fatty acid content on a wet weight basis, with oleic acid being the most abundant at 87.65 mg/g wet weight. Among saturated fatty acids, palmitic acid had the highest content. Pothoven, et al. [33] reported that palmitic, stearic, and oleic acids were predominant fatty acids in the omental adipose tissue of steers, although their contents varied with the size of the animals. Similarly, Mushi, et al. [34] found that the aforementioned fatty acids were abundant in the omental fat of male Small East African goats. In dairy cows, Hostens, et al. [35] observed similar results, reporting palmitic, stearic, and oleic acid contents of 24.7, 26.6, and 31.1 g/100 g fatty acids, respectively, in omentum tissue. The high oleic and palmitic acid content in bovine omentum underscores its potential as a source of recoverable lipid for human milk fat substitutes through enzymatic modification, where oleic acid can be incorporated at the sn-1,3 position and palmitic acid into the sn-2 position of triacylglycerols, thereby replicating the structural characteristics of human milk fat.

### 3.2. Collagen Yield, Degree of Hydrolysis, Free Amino Acid, and Protein Content

Yield of ASC and collagen extracted from the residue of bovine omentum after ASC extraction with the aid of Protana^®^ Prime at different concentrations is given in Table 3. The yield of ASC was 3.98% (based on the wet weight of bovine omentum). ASC primarily represents weakly cross-linked collagen fractions that are readily solubilized under acidic conditions due to protonation of charged amino acid residues and disruption of intermolecular hydrogen bonding. Following the extraction of the ASC, enzymatic treatment of the residue indicated that increasing the concentration of Protana^®^ Prime led to a substantial improvement in the yield of collagen, rising steadily from 4.98% at 1.0% (*w*/*w*) to 11.15% at 10% (*w*/*w*) (*p* < 0.05). This trend suggests a positive correlation between enzyme concentration and extraction efficiency. The highest yields were obtained when Protana^®^ Prime was used at 7.5% and 10% with no significant difference between these two levels (*p* > 0.05), indicating that further increases in enzyme concentration may not significantly enhance yield. Protana^®^ Prime enhanced collagen recovery from the residue by selectively cleaving non-collagenous proteins and partially hydrolyzing the telopeptide regions of collagen fibrils, thereby increasing solubility without fully disrupting the triple-helical structure.

With the exception of elastin, collagen is the only protein known to contain hydroxyproline [31]. Therefore, hydroxyproline is commonly used as a marker for determining collagen content. ASC from bovine omentum had a hydroxyproline content of 38.65 mg/g, while other samples varied between 27.08 and 35.04 mg/g (Table 3). Like yield, hydroxyproline content also increased with an increase in Protana^®^ Prime concentration (*p* < 0.05) from 27.08 mg/g at 1.0% to 33.63 mg/g at 7.5%. However, hydroxyproline content remained constant between 2.5% and 5% or 5% and 7.5% Protana^®^ Prime (*p* > 0.05), indicating that additional yield does not contain more collagen and may consist of soluble elastin. The highest hydroxyproline content of 35.04 mg/g at 10% Protana^®^ Prime. The high content of hydroxyproline content at higher enzyme concentration might be due to the hydrolysis of the telopeptide region by the enzyme, leading to increased solubilization and extraction yield. Mostly, pepsin was reported to cleave specifically at the telopeptide region of collagen without compromising the integrity of the triple helix. The enhanced extraction efficiency is further supported by an increase in DH with an increase in enzyme concentration (*p* < 0.05; Table 4). Moreover, free amino acid content was increased from 0.17 mM L-leucine equivalent at the lowest enzyme concentration (1%) to 1.41 mM L-leucine equivalent at the highest level tested (10%) (*p* < 0.05). Similarly, the protein content of the supernatant rose from 70.73 mg/g wet sample to 102.93 mg/g wet sample as the enzyme concentration increased (*p* < 0.05).

### 3.3. Effect of pH and NaCl on the Solubility of Collagen Samples

**Figure 2:**
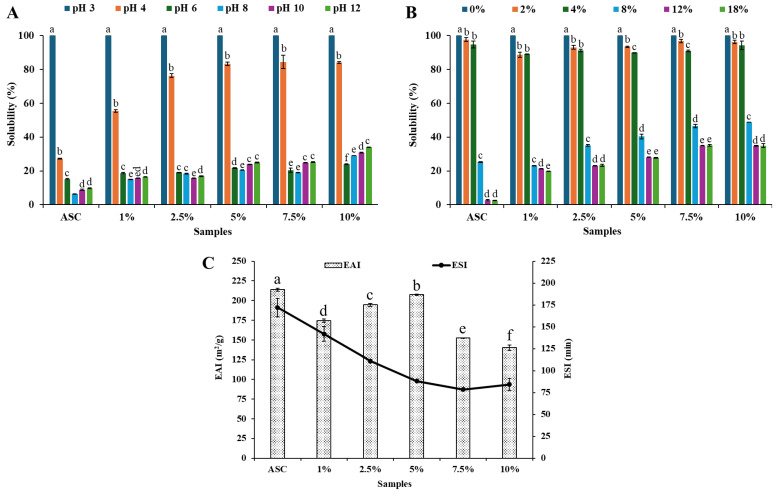
pH solubility (**A**), NaCl solubility (**B**), and emulsifying activity index (**C**) of acid-soluble collagen (ASC) from bovine omentum and collagen extracted from residue obtained after recovery of ASC using Protana^®^ Prime at different concentrations (1–10%, *w*/*w*). Different lowercase letters above the bar indicate significant differences within the same treatment at different pH and NaCl concentrations (*p* < 0.05).

The effects of pH and NaCl concentrations on the solubility of collagen are depicted in Figure 2A and Figure 2B, respectively. Irrespective of treatment, the highest solubility of collagen from the bovine omentum was found at pH 3. Pezeshk, et al. [36] also observed the highest solubility of native collagen from tuna by-products between pH 2 and 3. Generally, acid disrupts the intermolecular bonds and cross-links that hold the collagen fibres together. The free protons from the acid protonate the amino acids on the collagen chains, creating a net positive charge that causes electrostatic repulsion between the collagen molecules, promoting their separation and dissolution in the solution. A sharp decrease in the solubility was observed when the pH was above 4, and all the collagen samples had their lowest solubility between pH 4 and 6, indicating that the pI of the collagen samples was approximately pH 5. Further, the solubility of the collagen samples slightly increased at pH 10 and then remained steady until pH 12. Proteins acquire an overall negative charge at pH values above their isoelectric point (pI) and a positive charge below it, resulting in electrostatic repulsion among protein molecules. Such repulsion promotes greater interaction between the charged proteins and surrounding water molecules, thereby enhancing solubility. However, across all collagen samples, solubility in alkaline pH conditions was lower than that observed under acidic conditions, a characteristic commonly reported for collagen [37,38].

The solubility of collagen samples was influenced by a change in NaCl concentrations (Figure 2B). All samples showed the highest solubility in the absence of NaCl (*p* < 0.05). The solubility of collagen samples was decreased with the addition of NaCl, irrespective of treatment (*p* < 0.05). The solubility of all samples was drastically reduced at 8% NaCl (*p* < 0.05). Zhao, et al. [39] reported that the solubility of beef tendon type I collagen decreases with increasing NaCl concentration. Similarly, Zhou, et al. [40] observed a gradual reduction in the solubility of acid-soluble collagen extracted from silver carp swim bladder as NaCl concentration increased. This reduction in solubility can be attributed to the ‘salting-out’ effect, i.e., when NaCl is added to protein solution, the chloride ions (Cl^−^) tend to bind to positively charged sites on the protein surface due to having high charge density, reducing the overall net charge and weakening electrostatic repulsion among protein molecules. As a result, protein aggregation is promoted. Additionally, the increased ionic strength causes salt ions to compete with proteins for water molecules, enhancing hydrophobic interactions and interchain associations, which further facilitate protein precipitation and reduce solubility. Moreover, solubility was not changed between 12% and 18% NaCl, regardless of sample (*p* > 0.05). Interestingly, collagen extracted with the aid of Protana^®^ Prime from bovine omentum residue obtained after ASC extraction showed higher solubility than ASC at NaCl concentrations of 8% or higher (*p* < 0.05), irrespective of enzyme concentration (*p* < 0.05). Furthermore, the solubility of Protana^®^ Prime-assisted extracted collagen increased with higher enzyme levels at NaCl concentrations of 8% or higher (*p* < 0.05). This enhanced solubility may be attributed to the enzyme’s partial hydrolysis effect, which produces shorter peptide chains with more exposed hydrophilic groups. These structural modifications improve water–protein interactions and reduce aggregation tendency, allowing the enzyme-aided collagen to maintain higher solubility even under elevated salt concentrations.

### 3.4. Emulsifying Properties of Collagen Samples

Emulsion activity index (EAI) and emulsifying activity index (ESI) of ASC from bovine omentum and Protana^®^ Prime assisted extracted collagen from bovine omentum residue obtained after ASC extraction is presented in Figure 2C. EAI is the ability of a protein to adsorb to the surface of lipid particles in an emulsification system [41] and ESI is the ability of a protein to maintain the stability of the emulsion without separation of the water and oil phases. Among all samples, ASC exhibited the highest EAI and ESI (*p* < 0.05). This indicates that native collagen structure possesses superior emulsifying capacity, likely due to its intact triple-helical conformation and high contents of hydrophobic amino acids such as Ala, Pro, Phe, Met, Val, Leu, and Ile, with charged residues (Asp, Glu, Lys, Arg), which facilitates efficient adsorption at the oil-water interface. EAI values for enzyme-assisted extracted collagen were lower than ASC, irrespective of the sample (*p* < 0.05). The result indicated that Protana^®^ Prime might cleave the telopeptide region, leading to partial hydrolysis and lowering of hydrophobic amino acids, which in turn lowers the emulsifying activity. Similarly, the ESI value was higher for ASC as compared to enzyme-assisted extracted collagen (*p* < 0.05). This might be due to rearrangement in the triple helix or a change in protein conformation during enzymatic treatment, resulting in an inability to form a strong and cohesive interfacial film around oil droplets, thus causing coalescence and creaming. Moreover, EAI values gradually increased with increasing concentrations of Protana^®^ Prime up to 5% (*p* < 0.05). The higher EAI might be attributed to partial cleavage of telopeptides and other non-helical regions, which exposed buried hydrophobic residues such as Phe, Met, Ala, and Pro. The exposure of these residues might enhance surface hydrophobicity and molecular flexibility, thereby improving emulsification properties. Enzyme-assisted extracted collagen samples displayed a gradual decline in ESI with increasing Protana^®^ Prime concentrations, with values of 141.84, 120.49, 118.23, 116.71, and 112.28 min for 1%, 2.5%, 5%, 7.5%, and 10% enzyme, respectively (*p* < 0.05). The results indicated that enzymatic treatment generates smaller peptides that can rapidly adsorb to the oil–water interface, enhancing initial emulsification (high EAI), but they may form thinner, less cohesive interfacial films that are less resistant to coalescence or flocculation during storage. Additionally, the increased presence of low-molecular-weight peptides might reduce interfacial viscosity and elasticity, weakening the protective barrier surrounding oil droplets. As a result, emulsions become more prone to instability over time, reflected by the lower ESI values at higher Protana^®^ Prime concentrations. When the enzyme concentration was higher than 5%, EAI values were decreased (*p* < 0.05). Although no significant difference was observed between the EAI values of collagen extracted with 5% and 7.5% Protana^®^ Prime (*p* > 0.05). A further increase in Protana^®^ Prime to 10% significantly decreased EAI (*p* < 0.05). This reduction most likely occurs because extensive hydrolysis at higher enzyme levels disrupts the collagen triple helix structure, producing smaller peptide fragments with reduced amphiphilic properties that are less effective at stabilizing emulsions. Generally, moderate enzyme treatment can enhance the emulsifying properties of the protein, whereas high enzyme concentration can negatively affect the emulsifying capacity of the resulting protein [42]. Moreover, Zhang, et al. [43] stated that lower DH showed a better emulsifying property than those with a higher DH.

### 3.5. FTIR Spectra of Collagen Samples

**Figure 3:**
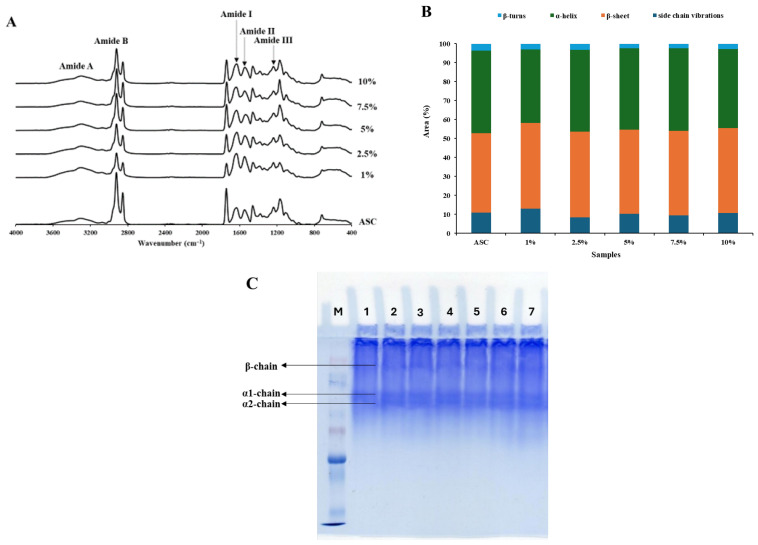
FTIR spectra (**A**), secondary structure analysis (**B**), and protein patterns (**C**) of acid-soluble collagen (ASC) from bovine omentum and collagen extracted from residue obtained after recovery of ASC using Protana^®^ Prime at different concentrations (1–10%, *w*/*w*). M: Marker; Lanes 2: ASC and 3-7: Collagen extracted from residue obtained after recovery of ASC using Protana^®^ Prime at 1, 2.5, 5, 7.5, and 10% (*w*/*w*), respectively.

FTIR spectra of all collagen samples are illustrated in Figure 3A. In general, collagen derived from various sources exhibits characteristic absorption peaks corresponding to amide A (N-H stretching vibration), amide B (asymmetrical CH_2_ stretching), amide I, II, and III, typically observed at 3328 cm^−1^, 2919 cm^−1^, 1700–1600 cm^−1^, 1600–1500 cm^−1^, and 1310–1175 cm^−1^, respectively [44,45]. When the N-H group of a peptide is involved in a hydrogen bond, this band shifts to lower frequencies. A similar pattern was observed for ASC extracted from bovine omentum in this study (Figure 3A). ASC spectrum displayed distinct peaks for amide A and amide B at 3304 cm^−1^ and 2917 cm^−1^, respectively. The amide I band was at 1637 cm^−1^, associated with the stretching vibration of C=O and from both C-N stretching and NH bending vibrations. The aforementioned band is a sensitive marker of the polypeptide secondary structure. ASC possessed amide II (C-N stretching and NH bending vibrations) and III (C-N stretching, N-H bending, C-C stretching, and C-N bending) at 1550 cm^−1^ and 1239 cm^−1^. Moreover, FTIR spectra of collagen samples obtained from bovine omentum residue after ASC extraction exhibited similar peak assignments across all enzyme concentrations (Figure 3A). The characteristic Amide A and Amide B bands were consistently observed at 3298 cm^−1^ and 2920 cm^−1^, respectively, while the amide I (1637 cm^−1^), amide II (1550 cm^−1^), and amide III (1239 cm^−1^) bands were present in all samples. Despite this similarity, the overall band intensities were lower than those observed in ASC, indicating a reduction in molecular order and potential alteration of secondary structure. With increasing enzyme concentration, the intensities of the amide I and amide II bands showed a gradual enhancement, suggesting partial unfolding and relaxation of the collagen triple-helical conformation induced by enzymatic treatment. However, the intensities were lower than those of ASC. Moreover, the triple-helical structure of collagen was reconfirmed by the peak ratio between amide III and the 1450 cm^−1^ bands [44]. According to Guzzi Plepis, et al. [46], an absorption ratio of approximately 1 between the amide III and the 1450–1454 cm^−1^ band indicates that the triple helical structure is intact. Ratios of 1.02 and 0.98 to 1.05 were obtained for ASC and enzyme-assisted extracted collagen samples, respectively, indicating the triple helical structures were maintained.

FTIR analysis through deconvolution of the amide I region (1600–1700 cm^−1^) revealed secondary structure components of ASC and collagen extracted from bovine omentum residue at different Protana^®^ Prime concentrations. The relative area percentages of the amide I sub-bands are presented in Figure 3B. In ASC from bovine omentum, characteristic peaks were observed at approximately 1691 cm^−1^ (β-turn), 1657 cm^−1^ (α-helix/triple helix), 1628 cm^−1^ (β-sheet), and 1608 cm^−1^ (side-chain vibrations). The α-helix/triple-helix and β-sheet components accounted for 43.49% and 44.08% of the total amide I area, respectively, whereas the side chain and β-turn structures contributed 8.86% and 3.57%. A similar spectral profile was observed in collagen extracted from bovine omentum residue obtained after ASC using Protana^®^ Prime, where α-helix/triple-helix and β-sheet structures remained dominant, indicating overall preservation of the native triple-helical conformation. With increasing enzyme concentration, a slight increase in the α-helix/triple-helix band is observed, suggesting exposure of helical structure by removing the telopeptide region of collagen and insoluble protein. This partial hydrolysis improves solubility and yield while maintaining the integrity of the helical structure. However, enzyme at 10% led to a decrease in the α-helix/triple-helix content, accompanied by an increase in side chain and β-turn content, indicating fragmentation, partial denaturation, and aggregation of collagen chains. Thus, the FTIR spectra confirm that Protana^®^ prime up to 7.5%, promoting efficient collagen extraction without compromising its native triple-helical structure, whereas above 7.5%, enzymatic action results in structural degradation.

### 3.6. Amino Acid Content and Composition of Collagen Samples

The amino acid content and composition of the collagen samples from bovine omentum, expressed as mg/g dry weight and amino acid residues/1000 amino acid residues, are given in Table 5. The total amino acid content in ASC was 566.64 mg/g, and Protana^®^ Prime-treated samples varied from 419.77 to 625.64 mg/g. Tryptophan was excluded from quantification due to its high susceptibility to degradation during acid digestion. Nevertheless, it should be noted that tryptophan can be recovered using microwave-assisted alkaline hydrolysis under conditions comparable to acid hydrolysis. In ASC from bovine omentum, glycine was the major amino acid (318 residues/1000 amino acid residues, 31.8%), followed by alanine (159 residues/1000 amino acid residues), glutamic acid/glutamine (91 residues/1000 amino acid residues), phenylalanine (58 residues/1000 amino acid residues), proline (51 residues/1000 amino acid residues), and hydroxyproline (46 residues/1000 amino acid residues). Glycine accounts for approximately one-third of all amino acid residues in collagen and appears at regular intervals of every third position, with the exception of the telopeptide regions. [47,48]. Su, et al. [49] reported 288 glycine residues/1000 amino acid residues in type I calf skin. Also, Li and Wu [50] reported 30–35% glycine content in type I to type VII collagen. Moreover, collagen extracted from striped catfish skin and Salmon skin collagen also had approximately 30% glycine content of total amino acid [51,52]. The high content of glycine, alanine, and proline reflects an intact collagen structure [53]. Similarly, glycine was approximately 30% of the total amino acid content in all collagen samples extracted using Protana^®^ Prime. Moreover, all samples contain essential amino acids, approximately 30% of the total amino acids. Moreover, it was observed that the amino acid content of collagen samples increased with increased Protana^®^ Prime concentration. Enzyme-assisted extraction might facilitate hydrolysis of the peptide bond between insoluble collagen and soluble collagen, thereby enhancing collagen solubility and resulting in higher amino acid content. Additionally, the enzyme may promote collagen solubilization by disrupting intermolecular cross-links. This trend was further supported by the observed increase in yield with increased enzyme concentration. The highest amino acid content was achieved when Protana^®^ Prime at 7.5% was used for extraction. However, a further increase to 10% led to a decline in amino acid content, likely due to excessive hydrolysis that generates free amino acid and soluble protein in higher content.

### 3.7. Protein Pattern of Collagen Samples

Protein patterns of the ASC and Protana^®^ Prime-assisted collagen extracted from bovine omentum residue obtained by ASC extraction are shown in Figure 3C. All collagen samples contained α-chains (α1 and α2) along with β-chains, which are dimers formed from the α-chains. The α1 and α2 bands of all samples had similar molecular weights (MW), approximately 120 kDa and 110 kDa, respectively. The β-chain with MW of 203 kDa and other high-molecular-weight components (HMC) were also observed. The ratio of α1 to α2 chains was about 2:1 in all the samples, suggesting type I collagen. Moreover, the protein patterns demonstrated that the enzymatic treatment did not affect the polypeptide pattern of the collagens, in terms of MW or intensity of each band, irrespective of concentration. Moreover, there were no low MW proteins noticed in the enzyme-assisted extracted collagen samples, indicating intact triple helix aligns with the FTIR discussion.

## 4. Conclusions

Bovine omentum, an underutilized by-product from beef processing, was successfully valorized as a novel source of type I collagen. Pre-treatment using NaCl and NaOH to remove non-collagenous protein and defatting using isopropanol was performed to minimize hindrance in the extraction, thereby achieving enhanced yield. pH 5 was isoelectric point of collagen. Enzyme-assisted extraction enhanced overall collagen recovery by valorisation of the residue obtained after ASC extraction. Both acid-soluble and enzyme-extracted collagens had amides I, II, and III, which are characteristic of collagen protein as revealed by FTIR spectroscopy and retained triple helical structure as per secondary structure analysis. Among the samples, ASC exhibited superior solubility and emulsifying properties, likely due to hydrophobic amino acid residues. Collagen extracted using Protana^®^ Prime exhibited enhanced emulsifying properties with increasing enzyme concentration, which was attributed to an increase in hydrophobic amino acid content. Glycine, proline, and hydroxyproline were major amino acids in collagen samples. Overall, the results demonstrate that bovine omentum serves as a sustainable and functional alternative source of collagen for various food and industrial applications.

## Figures and Tables

**Table 1:** Proximate composition of bovine omentum.

Parameters	Values on Wet Weight Basis (%)
Moisture	35.25 ± 2.51
Crude protein	18.79 ± 1.10
Crude lipid	42.14 ± 2.90
Carbohydrates	3.77 ± 0.71
Ash	0.06 ± 0.02

Data is presented in mean ± SD (*n* = 5).

**Table 2:** Amino acid and fatty acid composition of bovine omentum.

Amino Acid	Amount (mg/g Wet Weight)	Fatty Acid	Amount (mg/g Wet Weight)
Glycine	43.10 ± 4.62	***SFA***	
Phenylalanine	22.80 ± 3.39	C16:0 Palmitic acid	72.97 ± 6.50
Alanine	22.29 ± 1.93	C18:0 Stearic acid	43.35 ± 3.80
Glutamic acid	22.41 ± 1.21	C14:0 Myristic acid	9.64 ± 2.35
Methionine	15.62 ± 0.88	C17:0 Heptadecanoic acid	2.64 ± 0.70
Arginine	12.81 ± 1.02	C15:0 Pentadecanoic acid	1.26 ± 0.31
Lysine	11.57 ± 0.29	C20:0 Arachidic acid	0.43 ± 0.08
Hydroxyproline	10.83 ± 0.45	***MUFA***	
Aspartic acid	8.95 ± 0.50	C18:1n9c Oleic Acid	87.65 ± 3.63
Serine	6.25 ± 0.29	C16:1n7c Palmitoleic acid	1.37 ± 0.15
Histidine	5.77 ± 0.20	C17:1n7c Heptadecenoic acid	1.35 ± 0.39
Isoleucine	5.59 ± 1.10	C14:1n5c Myristoleic acid	1.85 ± 0.49
Threonine	5.22 ± 0.19	***PUFA***	
Proline	3.88 ± 0.28	C18:2n6c Linoleic acid	2.20 ± 0.49
Valine	2.97 ± 0.31	C18:3n4c 9,11,14-Octadecatrienoic acid	0.99 ± 0.03
Cysteine	1.53 ± 0.24	C18:3n3 alpha-Linolenic acid	0.58 ± 0.07
Tyrosine	0.90 ± 0.08	C18:2n6t Linolelaidic acid	0.15 ± 0.03
Leucine	0.51 ± 0.07	C20:3n6c 8,11,14-Eicosatrienoic acid	0.13 ± 0.03
EAA	70.04 ± 4.02	C20:4n6c Arachidonic acid	0.13 ± 0.01
NEAA	131.42 ± 11.50	C20:5n3c 5,8,11,14,17-Eisopentaenoic acid	0.02 ± 0.00
TAA	202.98 ± 12.22	C22:6n3c 4,7,10,13,16,19 Docosahexaenoic acid	0.01 ± 0.00

Data were presented as mean ± SD (*n* = 3). EAA: Essential amino acid; NEAA: Non-essential amino acid; TAA: Total amino acid; SFA: Saturated fatty acids; MUFA: Monounsaturated fatty acids; PUFA: Polyunsaturated fatty acids.

**Table 3:** Collagen yield and hydroxyproline content of collagen samples recovered from bovine omentum using acid or enzymatic (Protana^®^ Prime) hydrolysis.

Treatment	Yield (%)	Hydroxyproline content (mg/g Sample)
ASC	3.98 ± 0.09 ^e^	38.65 ± 0.67 ^a^
E 1%	4.98 ± 0.13 ^d^	27.08 ± 0.54 ^e^
E 2.5%	5.29 ± 0.05 ^c^	32.56 ± 0.61 ^d^
E 5%	7.07 ± 0.06 ^b^	33.65 ± 0.56 ^dc^
E 7.5%	11.13 ± 0.09 ^a^	33.63 ± 0.62 ^c^
E 10%	11.15 ± 0.03 ^a^	35.04 ± 0.53 ^b^

Data were presented as mean ± SD (*n* = 3). Different lowercase superscripts indicate a significant difference between different treatments (*p* < 0.05). ASC: Acid-soluble collagen, E: Enzymatic hydrolysis using Protana^®^ Prime.

**Table 4:** Degree of hydrolysis, free amino acid, and protein content of bovine omentum residue obtained after extraction of acid-soluble collagen extraction.

Protana^®^ Prime (%, *w*/*w*)	Degree of Hydrolysis (%)	Free Amino Acid Content (mM L-Leucine Equivalent)	Protein Content (mg/g wet Weight)
1	2.50 ± 0.11 ^a^	0.17 ± 0.01 ^e^	70.73 ± 1.63 ^a^
2.5	2.78 ± 0.37 ^a^	0.44 ± 0.01 ^d^	72.41 ± 1.57 ^a^
5	4.60 ± 0.22 ^b^	0.62 ± 0.02 ^c^	87.21 ± 5.07 ^b^
7.5	5.23 ± 0.30 ^c^	0.84 ± 0.01 ^b^	97.19 ± 2.76 ^c^
10	5.48 ± 0.19 ^d^	1.41 ± 0.01 ^a^	102.93 ± 2.74 ^d^

Data were presented as mean ± SD (*n* = 3). Different lowercase superscripts indicate a significant difference between different treatments (*p* < 0.05).

**Table 5:** Amino acid composition (mg/g dry weight) of collagen samples recovered from bovine omentum using acetic acid or Protana^®^ Prime hydrolysis.

Amino Acid	ASC	1%	2.5%	5%	7.5%	10%
Aspartic acid	21.63 ^a^ (32)	17.63 ^c^ (37)	19.48 ^b^ (30)	15.01 ^e^ (24)	14.91 ^e^ (20)	16.49 ^d^ (26)
Glutamic acid	67.74 ^a^ (91)	45.63 ^e^ (86)	57.16 ^b^ (80)	45.93 ^e^ (66)	51.93 ^c^ (61)	50.24 ^d^ (71)
Serine	19.39 ^a^ (37)	12.66 ^e^ (34)	15.86 ^c^ (31)	13.94 ^d^ (28)	16.89 ^b^ (28)	15.55 ^c^ (31)
Histidine	10.39 ^a^ (13)	10.18 ^a^ (18)	10.30 ^a^ (14)	9.92 ^b^ (14)	6.74 ^c^ (8)	ND (ND)
Arginine	43.96 ^a^ (50)	20.61 ^e^ (33)	35.95 ^c^ (43)	12.37 ^f^ (15)	36.88 ^b^ (37)	32.74 ^d^ (39)
Glycine	120.55 ^b^ (318)	71.87 ^f^ (265)	106.42 ^e^ (295)	109.92 ^d^ (313)	142.94 ^a^ (331)	115.98 ^c^ (322)
Threonine	12.16 ^a^ (20)	9.41 ^c^ (22)	11.48 ^b^ (20)	11.51 ^b^ (21)	12.41 ^a^ (18)	11.90 ^b^ (21)
Alanine	71.74 ^c^ (159)	44.75 ^e^ (139)	72.64 ^c^ (170)	67.23 ^d^ (162)	108.14 ^a^ (211)	74.81 ^b^ (174)
Tyrosine	6.83 ^d^ (7)	9.49 ^c^ (15)	21.49 ^a^ (24)	10.22 ^b^ (12)	5.34 ^e^ (5)	7.37 ^d^ (8)
Hydroxyproline	30.43 ^a^ (46)	24.34 ^d^ (52)	25.56 ^c^ (41)	25.50 ^c^ (42)	25.01 ^c^ (33)	26.02 ^b^ (41)
Valine	5.49 ^a^ (9)	4.33 ^c^ (10)	3.20 ^b^ (6)	4.02 ^b^ (7)	2.46 ^d^ (4)	3.37 ^c^ (6)
Methionine	35.66 ^d^ (47)	27.41 ^e^ (51)	42.10 ^c^ (59)	46.49 ^b^ (66)	53.38 ^a^ (62)	41.76 ^c^ (58)
Cysteine	2.28 ^b^ (4)	2.40 ^b^ (6)	1.81 ^c^ (3)	9.07 ^a^ (15)	1.94 ^c^ (3)	2.37 ^b^ (4)
Phenylalanine	48.15 ^f^ (58)	57.31 ^d^ (97)	49.75 ^e^ (63)	59.70 ^b^ (79)	61.28 ^a^ (64)	58.10 ^c^ (74)
Proline	29.71 ^c^ (51)	25.59 ^e^ (62)	28.18 ^d^ (51)	33.06 ^a^ (62)	33.03 ^a^ (50)	31.14 ^b^ (57)
Lysine	28.36 ^e^ (38)	19.82 ^f^ (38)	29.06 ^d^ (41)	29.75 ^c^ (44)	35.61 ^a^ (42)	30.38 ^b^ (43)
Isoleucine	9.59 ^b^ (15)	9.94 ^a^ (21)	9.99 ^a^ (16)	9.65 ^b^ (16)	9.67 ^b^ (13)	10.16 ^a^ (16)
Leucine	2.70 ^e^ (4)	6.39 ^c^ (14)	8.17 ^a^ (13)	8.32 ^a^ (14)	7.08 ^b^ (9)	4.85 ^d^ (8)
EAA	152.49 ^e^	144.79 ^f^	164.04 ^c^	179.35 ^b^	188.63 ^a^	160.53 ^d^
NEAA	411.977 ^b^	272.57 ^f^	382.74 ^c^	333.18 ^e^	435.07 ^a^	370.33 ^d^
TAA	566.64 ^b^	419.77 ^e^	548.59 ^c^	521.61 ^d^	625.64 ^a^	533.24 ^d^

Data are presented as mean. In parathensis, amino acid residues/1000 amino acid residues are given. Different lowercase superscripts indicate a significant difference between different treatments (*p* < 0.05). ASC: Acid-soluble collagen; EAA: Essential amino acid; NEAA: Non-essential amino acid; TAA: Total amino acid; ND: Not detected; 1–10% (*w*/*w*): Protana^®^ Prime.

## Data Availability

The original contributions presented in this study are included in the article. Further inquiries can be directed at the corresponding author.
